# Distribution of emergency operations and trauma in a Swedish hospital: need for reorganisation of acute surgical care?

**DOI:** 10.1186/1757-7241-20-66

**Published:** 2012-09-17

**Authors:** Fawzi al-Ayoubi, Helen Eriksson, Pär Myrelid, Conny Wallon, Peter Andersson

**Affiliations:** 1 Unit for Acute Care Surgery and Trauma, Department of Surgery, Linköping University Hospital, S-581 85, Linköping, Sweden

**Keywords:** Acute care surgery, Trauma, Centralization, Subspecialisation

## Abstract

**Background:**

Subspecialisation within general surgery has today reached further than ever. However, on-call time, an unchanged need for broad surgical skills are required to meet the demands of acute surgical disease and trauma. The introduction of a new subspecialty in North America that deals solely with acute care surgery and trauma is an attempt to offer properly trained surgeons also during on-call time. To find out whether such a subspecialty could be helpful in Sweden we analyzed our workload for emergency surgery and trauma.

**Methods:**

Linköping University Hospital serves a population of 257 000. Data from 2010 for all patients, diagnoses, times and types of operations, surgeons involved, duration of stay, types of injury and deaths regarding emergency procedures were extracted from a prospectively-collected database and analyzed.

**Results:**

There were 2362 admissions, 1559 emergency interventions; 835 were mainly abdominal operations, and 724 diagnostic or therapeutic endoscopies. Of the 1559 emergency interventions, 641 (41.1%) were made outside office hours, and of 453 minor or intermediate procedures (including appendicectomy, cholecystectomy, or proctological procedures) 276 (60.9%) were done during the evenings or at night. Two hundred and fifty-four patients were admitted with trauma and 29 (11.4%) required operation, of whom general surgeons operated on eight (3.1%). Thirteen consultants and 11 senior registrars were involved in 138 bowel resections and 164 cholecystectomies chosen as index operations for standard emergency surgery. The median (range) number of such operations done by each consultant was 6 (3–17) and 6 (1–22). Corresponding figures for senior registrars were 7 (0–11) and 8 (1–39).

**Conclusion:**

There was an uneven distribution of exposure to acute surgical problems and trauma among general surgeons. Some were exposed to only a few standard emergency interventions and most surgeons did not operate on a single patient with trauma. Further centralization of trauma care, long-term positions at units for emergency surgery and trauma, and subspecialisation in the fields of emergency surgery and trauma, might be options to solve problems of low volumes.

## Background

Subspecialisation in what has always been called general surgery has in most countries (including Sweden) been carried further than ever during the past decade resulting in breast, endocrine, colorectal, and upper gastrointestinal (GI) surgery being almost independent entities. Vascular surgery has become a specialty on its own. This has resulted from, among other things, more strict regulation and the recording of actual hours worked. Carefully regulated use of compensation leave in accordance with the European Working Time Directive has resulted in there being even less time than before for traditional general surgical training [1]. It has become necessary to focus on a narrow field of surgery if sufficient theoretical and practical knowledge is to be acquired within a reasonable period of time. This must be maintained to meet the quality demands for surgical practice that are raised by the profession, the patients, and by health care providers for elective surgery.

Outside office hours, however, there is still a need for broad and varied surgical competence in dealing with acute illness and trauma. In Sweden the surgical profession has attempted to solve this by offering, for consultant surgeons on call, specific courses within each subspecialty that focus on emergency conditions; they also offer courses in trauma care. Elsewhere, for example in the USA and Canada, attempts to solve similar problems have resulted in the introduction of the new surgical subspecialty “acute care surgery” [2,3]. One of its prerequisites as a distinct specialty is the establishment of certain units within hospitals that care for patients with acute surgical conditions and trauma. Several surgical departments in Sweden have already introduced such units for acute care surgery and others intend to do so. So far the need for subspecialisation in acute care surgery and trauma, such as in North America, for surgeons staffing these units, has been discussed only casually in Sweden. To address the issue of whether such subspecialisation could be advantageous in Sweden we analyzed the surgical activities at the unit for Acute Care Surgery and Trauma (ACST), Department of Surgery, Linköping University Hospital, during one year.

## Methods

Data about all patients treated in the ACST unit from Jan.1^st^ to Dec.31^st^, 2010 included diagnoses, operations, duration of operation, surgeon involved, duration of stay, readmission, trauma, and death were extracted from a prospectively collected database including basic perioperative and postoperative information about all patients treated at the Department of Surgery. The study was a clinical quality-control study approved by the Head of the Department of Surgery, Linköping University Hospital, Linköping, Sweden. Descriptive data are given as number (%) without further statistical analysis. They were handled and analyzed on Statistical software version 9.0 (Statsoft Inc. Tulsa, OK, USA).

Linköping University Hospital is the only hospital that serves a population of 257 000 for emergency surgery and trauma. It also serves a further 835 000 as a secondary and tertiary referral centre, mainly for elective surgery and advanced trauma care such as neurological trauma or burns. The ACST has round-the-clock responsibility for all acute admissions and emergency operations and endoscopies in the surgical department, and provides acute consultations within the hospital and the emergency department, the latter mainly staffed by emergency physicians. It has at its disposal one dedicated operating theatre shared with obstetrics and gynaecology for acute cases, a surgical acute care ward with 28 beds, and an outpatient clinic two afternoons a week. All local and regionally referred trauma except for isolated neurological trauma are primarily dealt with in the ACST. Elective procedures are strictly separated from the activities of the ACST and dealt with by the units for colorectal, upper GI, and endocrine surgery.

During office hours the ACST is staffed by three, or sometimes four, senior registrars or consultants, usually one junior registrar, and one or two house officers all of whom are working exclusively in the unit. The permanent surgical staff consists of two full time consultants, one half-time consultant, two senior registrars, and one junior registrar with a long-term appointment. The remaining staff needed to cover vacations and compensation leave are met by a weekly rota of surgeons who rotate from other units within the department of surgery for either one or two weeks; this arrangement also aims to increase exposure to emergency surgery and trauma during the day to all surgeons in the department. Out of office hours there is a senior registrar on call in the hospital. A consultant who trained as a general surgeon, but specialized in either colorectal, upper-GI, or endocrine surgery, is on call outside the hospital and is prepared to intervene at short notice. Care for vascular emergencies is provided separately by vascular surgeons.

## Results

### Emergency surgery

During 2010 there were 2362 admissions (1175 (49.7%) of whom were men) to the ACST with a median age of 62 years (16–100). Median (range) duration of stay was 2 days (1–118). One-hundred and ninety-five patients were readmitted within 30 days of discharge, and 32 died during their stay of whom 19 had been operated on. The most common diagnosis was benign biliary disease requiring surgery or therapeutic ERCP (endoscopic retrograde cholangiopancreaticography) in 76.2% of cases. Proctologic disease was along with appendicitis the diagnosis where the largest part of patients was subjected to surgical intervention; 97.2% and 92.5% respectively. After trauma and non-specific abdominal pain, patients diagnosed with small bowel obstruction, pancreatitis or diverticular disease of the colon represented diagnoses where least part of the patients required intervention (Table 1).

**Table 1:** Most common diagnoses at ACST 2010

**Diagnoses**	**No. (%) of admissions**	**Total No. of main operations including reoperations**	**Total No.of endoscopies**	**Percentage of patients in need of surgical or endoscopic procedures**
Benign biliary disease	282 (12.0)	152	105	76.2
Trauma	254 (10.8)	29	0	11.4
Appendicitis	214 (9.1)	199	0	92.5
Abdominal pain	197 (8.3)	9	26	16.2
Colonic diverticulitis	166 (7.0)	19	70	39.2
Malignant or possibly malignant tumours	162 (6.8)	61	106	75.3
Acid related disease (oesophagitis, bleeding ulcer or perforation)	147 (6.2)	26	171	93.2
Small bowel obstruction	137 (5.8)	44	8	31.4
Postoperative complications (as cause of readmission)	104 (4.4)	29	35	59.6
Pancreatitis	97 (4.1)	18	24	39.2
Proctological disease	72 (3.0)	65	13	97.2
Abdominal hernia	57 (2.4)	44	0	78.9
Inflammatory bowel disease	36 (1.6)	12	10	63.8
Miscellaneous	361 (15.3)	105	139	47.6
Missing diagnoses	76 (3.2)	23	17	43.4
**Total**	**2362**	**835**	**724**	**50.7**

The ACST provided care for 126 patients who required treatment in the intensive care unit (ICU) with a median stay of 2 days (1–31); the cause was trauma in 44 cases. A total of 1559 emergency interventions were made, which consisted of 835 mainly abdominal operations and 724 diagnostic or therapeutic endoscopies. These included, for example, hemostasis, removal of polyps, or ERCP with sphincterotomy. Twenty patients (2.4%) were reoperated on within 30 days*.* The most common intervention was diagnostic endoscopy by means of upper-GI endoscopy or colonoscopy, and the most common operations were appendicectomy, cholecystectomy, and proctological examinations under anesthesia with interventions. A total of 1559 emergency interventions 641 (41.1%) were done out of office hours. Most common minor and intermediate operations (appendicectomy, cholecystectomy, and proctological interventions) were done during the evening or at night (between 1700 and 0800) (276/453, 60.9%) as opposed to major procedures such as colectomies or colonic resections, fewer of which were done during that time (33/80, 41.2%). Corresponding figures for therapeutic endoscopy during the evening or at night were 11 of 217 (5.1%), all of which were prompted by upper gastrointestinal bleeding and necessitated endoscopic hemostasis (Table 2).

**Table 2:** Most common invasive procedures at ACST 2010

**Operations**	**Office hours**	**Weekend**	**Evening**	**Night**	**Total No.**
	**0800-1700**	**0800-1700**	**1700-2400**	**0000-0800**	
Diagnostic endoscopy	422	41	31	13	507
Therapeutic endoscopy	189	17	7	4	217
Appendicectomy	33	12	91	78	214
Cholecystectomy	69	41	50	4	164
Proctological procedure	15	7	32	21	75
Colonic resection	27	5	18	14	64
Hernia repair	17	1	8	11	37
Reoperation for complication	15	2	13	6	36
Division of adhesions	11	2	10	4	27
Exploratory laparotomy	6	3	13	3	25
Stoma procedure	13	3	6	2	24
Gastroduodenal intervention	11	3	3	5	22
Subtotal colectomy	13	2	0	1	16
Small bowel resection	5	2	5	3	15
Diagnostic laparoscopy	4	2	3	5	14
Miscellaneous	68	16	11	7	102
**Total**	***918***	***159***	***301***	***181***	**1559**
	*(58.9%)*	*(10.2%)*	*(19.3%)*	*(11.6%)*	*(100%)*

Data are number of patients divided by time of day.

### Trauma

The trauma team was activated 181 times, and 254 patients were admitted (Table 1). The most common mechanism of injury was a fall (n = 126), and the second was traffic crashes (n = 69) followed by sport and leisure activities (n = 23), and assaults (n = 23). The vast majority of trauma-cases were the result of blunt trauma, only four injuries being penetrating ones caused by a knife or firearm. Of those admitted, 29 (11.4%) required a total of 40 operations. General surgeons were involved in only eight (3.1%) of these patients, specifically in exploratory laparotomy n = 5 (enterorrhaphy n = 2, splenectomy n = 1, and no abnormality found n = 2), thoracotomy n = 1 (no abnormality found), and cervical injury n = 2 (ligation of external and internal carotid arteries). The remaining interventions were covered by orthopedics, neurosurgery, or ear nose and throat consultants. Forty-four patients (17.3%) required intensive care and were treated under the care of the general surgeons for at least the first 24 hours according to the routines for multitrauma care at the hospital.

### Distribution of interventions among surgeons

**Figure 1:**
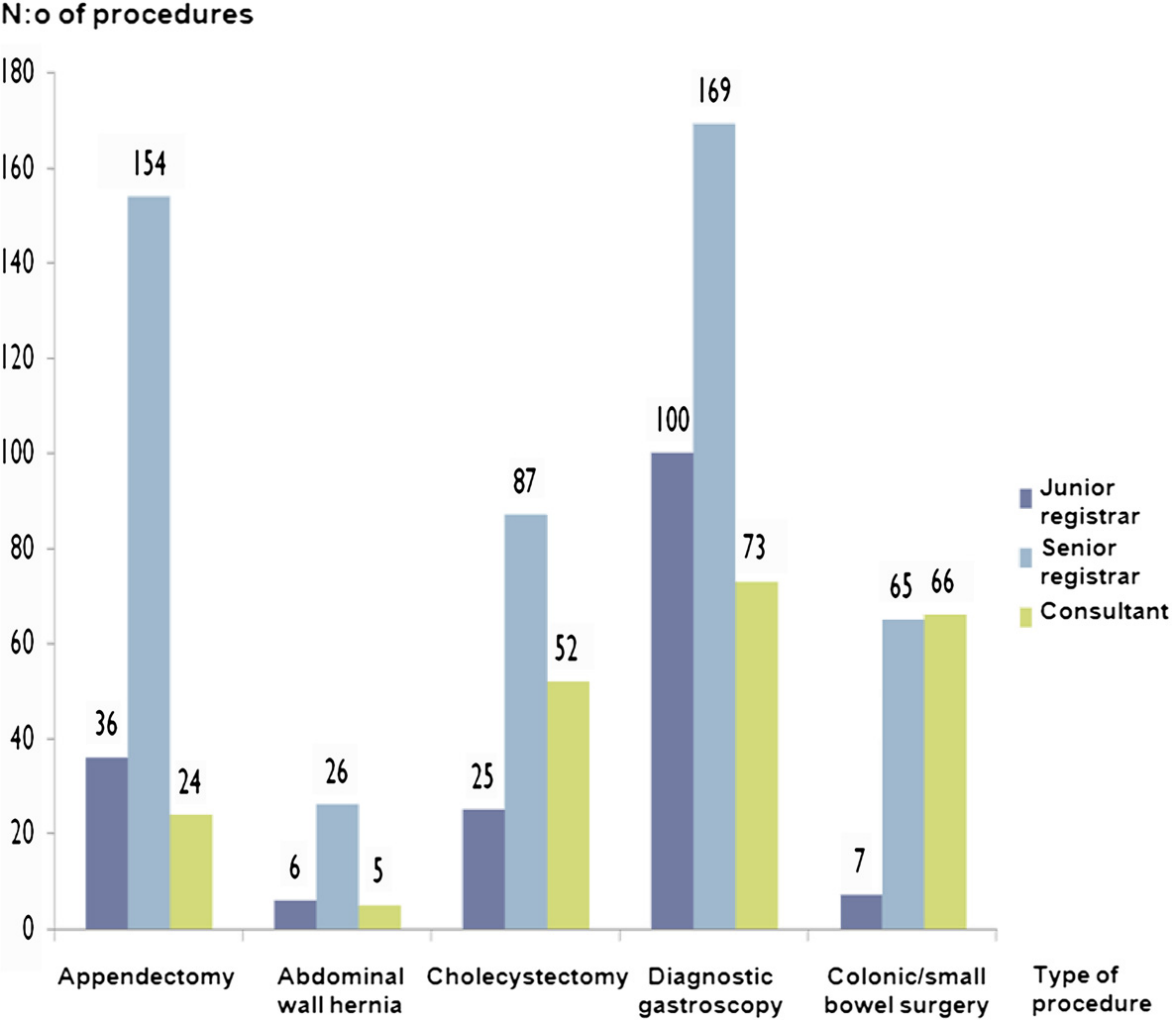
Some of the most common invasive surgical operations or endoscopic procedures done at the unit for Acute Surgery and Trauma during 2010 divided after the level of experience of the operating surgeon.

Most of the most common operations during office hours and while on call were done by either a senior registrar (n = 11) or a consultant (n = 13) as the main surgeon (diagnostic gastroscopy (70.8%), appendicectomy (83.2%), hernia repair (83.8%), cholecystectomy (84.8%), and colonic or small-bowel resection, including formation of a stoma (94.9%). The corresponding figures for the junior registrars in the department (n = 4) as main surgeon were 29.2%, 16.8%, 16.2%, 15.2% and 5.1%, respectively (Figure 1).

On call service outside the hospital and the resultant potential exposure to emergency surgery during on call time were equally divided during the year among 13 consultants in general surgery whose subspecialties were colorectal, upper-GI, and endocrine surgery. The commitment of time to emergency surgical interventions during office hours differed among consultants as a result of their variable rotational commitments at the ACST. Some of the consultants did not actually serve there at all, or only in a limited way, while one of them spent most of his time there. For common intermediate emergency interventions such as colonic or small-bowel resections, including stoma surgery (n = 138), or gallbladder surgery (n = 164), the median number of operations done by each individual consultant during the year (either as the main or assisting surgeon) was 6 (3–17) and 6 (1–22), respectively. The corresponding number for the 11 senior registrars on call in the hospital, of whom three spent their days in the ACST, was 7 (0–11) and 8 (1–39), respectively; the highest figures were those assigned to the ACST.

## Discussion

The current trend for general surgery in Sweden towards greater subspecialisation within units, and also towards a reduction in the number of facilities that provide acute health care, including the number of surgical departments, is clear [4]. If we focus more on excellence among surgeons, each one who works within a narrowly-defined elective surgical field (while at the same time being governed by stricter regulation of working hours specified by the European Union directives) has ever less time available to gain competence within the broad area of general surgery. Emergency surgery and traumatology, still the responsibility of most surgeons during their on call time, requires sufficient training in general surgery.

Our simple descriptive data about emergency surgery and the treatment of trauma during a single year at the University Hospital in Linköping show that more than half the patients have some form of intervention from a relatively large number of surgeons. More than 40% of emergency operations in our hospital are done out of office hours, commonly during evenings and nights when informal support from skilled colleagues may be hard to find. It can be claimed that most procedures are simple but this is not always true for, for example, intestinal resections, bleeding duodenal ulcers, or even cholecystectomies for acute cholecystitis. Ideally fewer patients should be operated on during the evening or night than is now current, almost 31% being treated. Only about half of them really need emergent operations, while the rest actually present day-time with less emergent indications, i.e. urgent, that can wait up to six hours or more. One of the reasons for the disproportion between night and day is the irrational nature of acute surgery which in the light of increasing demands for effective use of health-care resources results in difficulties to assign more than one operating theatre during the day. This ultimately delays surgery to inconvenient hours.

Most of the most common emergencies are dealt with by surgeons who are specialists in some subspecialty, but the median number of interventions is relatively limited. The dispersion among individual surgeons is substantial, as some do only a single or even no emergency interventions such as cholecystectomies or intestinal resections during an entire year. It is also noteworthy that the annual number of therapeutic endoscopies during the evening and night shifts is small, and even if the 17 cases that are done during the weekend day shifts are added, most of them to control bleeding, there is hardly more than one case/surgeon.

Another finding is that only one in 10 of the patients treated for trauma, even in a catchment area of almost 260 000 people, requires operation other than simple thoracic drainage, and only a fraction of these interventions is done by those who are seen traditionally as general surgeons. During a time as short as a single year, it might be possible that the varying numbers of interventions to some extent depend on natural fluctuations in the stream of incoming patients during specific time periods seen from the point of view of a single day, or even the whole year, but they are in the end more likely to be the result of the organization of emergency surgery and trauma. Given our existing organization it is quite clear that the exposure to emergency surgery and trauma can be quite limited.

Centralization and concentration of less common and more complex interventions for cancers such as those of the esophagus, pancreas, and rectum in designated units improves the results both in terms of complications and long-term survival [5-7]. A large volume of interventions by individual surgeons who deal with these types of diagnoses is associated with better results than those seen when surgeons deal with only a small number [8]. There are reasons to think that the same relations are likely to hold for emergency surgery, trauma, and acute endoscopy. This assumption is supported by data from designated emergency surgical care centers that have reported shorter postoperative recovery times and fewer postoperative complications for appendicitis and cholecystitis [9,10]. Consequently, regionalization of emergency operations to high-volume centers to improve results has previously been suggested [11].

As far as trauma management is concerned, it has been unambiguously established that mortality is lower at high-volume centers than at other centers [12]. Although Linköping University Hospital has a population of almost 260 000 in its primary catchment area and is, in a Swedish context, a large hospital, it cannot be regarded as a high-volume trauma centre from the reported number of cases treated. A case may therefore be made that further centralization of trauma care in Sweden that results in even larger catchment areas for trauma would lead to an improvement in quality, still given the relatively small absolute number of cases.

Emergency surgery and trauma care are mainly dealt with by surgeons out of office hours. The current European Working Time Directive that restricts work to 48 hours a week, including that out of office hours, means that emergency surgery encroaches on time set aside for elective surgery. This unfortunately results in competition between elective and emergency surgery, one of which will have an adverse impact on the other. During previous decades repeated reduction in working hours for registrars in the UK has been clearly shown to have a serious impact on the opportunities for surgical training [13]. Concerns have also been raised in Norway about the quality of training for surgical residents in the light of structural changes in the health care system [14]. Our data have shown that there is all too little participation in emergency surgery and endoscopy on the part of our junior registrars, and this supports the previous findings and concerns.

## Conclusions

In a Swedish university hospital setting there was an uneven distribution of exposure to acute surgical problems as well as trauma among surgeons. Some were exposed to only a few standard emergencies and most surgeons did not operate on a single patient with trauma. Residents were responsible for strikingly few emergency procedures. Dealing with acute surgery and trauma out of office hours calls for a multi-faceted knowledge of surgical approaches that is difficult to attain in the present system and will become even more difficult in the future. Further centralization of trauma care, long-term positions at units for emergency surgery and trauma and sub-specialization in the fields of emergency surgery and trauma might be solutions.

## Abbreviations

GI: Gastrointestinal; ACST: Acute care surgery and trauma; ERCP: Endoscopic retrograde cholangiopancreaticography; ICU: Intensive care unit.

## Competing interests

None of the authors have any competing interests.

## Authors’ contributions

FaA contributed to the conception and design and drafted the manuscript. HE acquired all data. PM analyzed the data and drafted the manuscript. CW contributed to the conception. PA contributed to conception and design, analyzed the data, and drafted the manuscript. All authors revised the manuscript and approved the final version.
